# Using Mandatory Sales Reports to Monitor Same‐Day Alcohol Delivery Trends in New South Wales

**DOI:** 10.1111/dar.70142

**Published:** 2026-03-18

**Authors:** Nicholas Taylor, Kira Button, Michael Livingston, Amy Peacock, William Gilmore, Michala Kowalski

**Affiliations:** ^1^ National Drug Research Institute Curtin University Perth Australia; ^2^ Burnet Institute Melbourne Australia; ^3^ School of Psychology Deakin University Geelong Australia; ^4^ National Drug and Alcohol Research Centre UNSW Sydney Sydney Australia

**Keywords:** alcohol, Australia, delivery, policy, sales data

## Abstract

**Introduction:**

Industry reports suggest that same‐day alcohol delivery service usage has increased substantially in recent years. In 2021, the New South Wales (NSW) government established a framework to reduce the risks associated with same‐day deliveries, which included mandatory sales reports from retailers. Using this data, this study aims to quantify the nature of the same‐day delivery market across NSW.

**Methods:**

Six‐monthly aggregate data from July 2021 to June 2024 were obtained on the volume of beer, wine and spirits self‐reported by alcohol retailers as sold by same‐day delivery, by delivery postcode. Frequencies were used to examine the number of retailers, postcodes and amount of alcohol sold in each reporting period. Heat maps were used to examine sales per capita by postcode. Comparisons were also made with data extracted from publicly available industry reports and national consumption estimates.

**Results:**

Forty‐nine retailers reported sales, with four accountable for 89% of alcohol sold, and only 8 retailers consistently reporting across the entire period. Market share fluctuated substantially by retailer and liquor category. Statewide per capita consumption was 0.09 L of pure alcohol in 2021–22, 0.07 in 2022–23, and 0.08 in 2023–24. Comparison to industry data suggested that on average over a litre of pure alcohol was delivered per transaction.

**Discussion and Conclusion:**

NSW sales data has the potential to provide a unique insight into the nature of the same‐day delivery market. The current level of data aggregation limits its utility; a lack of compliance checking and anomalies in the data bring its validity into question.

## 1 Introduction

Internationally, the use of same‐day alcohol delivery services has risen considerably in recent years, contributing to a substantial increase in alcohol availability and accessibility [1, 2]. In Australia, online alcohol sales increased almost four‐fold in value from 2012 ($539 million) to 2022 ($2.1 billion) [3, 4]. The COVID‐19 pandemic conditions accelerated this growth, with several Australian jurisdictions amending liquor licencing regulations to permit businesses without off‐premise licences to sell alcohol via takeaway and delivery [5]. Following the easing of pandemic restrictions, many consumers continued use of these services, with increasing uptake primarily attributed to factors such as convenience, affordability and ease of access [6].

While same‐day alcohol delivery services provide convenience for consumers, they also present potential public health concerns [7]. Delivery may pose particular risks to people with alcohol use disorders, indeed two recent coronial hearings have found that high frequency home deliveries contributed to death from acute ethanol toxicity [8, 9]. Several studies have examined associations between same‐day alcohol delivery and drinking behaviour among the general population in Australia, highlighting a relationship with increased consumption and risky drinking patterns [10, 11, 12]. A large national cross‐sectional survey (*N* = 1158) found that 20% of respondents who had used online alcohol delivery services in the last 3 months used alcohol delivery to continue a drinking session, and rapid delivery (i.e., within 2 h of order) users were six times more likely to engage in hazardous drinking [10]. Another cross‐sectional study showed that high‐risk drinkers in Western Australia were almost 12 times more likely to receive alcohol deliveries while intoxicated compared to low‐risk drinkers [11]. Cross‐sectional survey data from the US has shown delivery service use to be associated with greater levels of alcohol consumption and negative consequences of drinking [13]. Qualitative research further suggests that ease of access and convenience associated with same‐day alcohol delivery contributes to increased overall consumption [6]. Collectively, these findings highlight the association between same‐day alcohol delivery and harmful drinking. However, the cross‐sectional design of these studies only provides a snapshot of service‐users at one point in time, limiting ability to examine long‐term trends in same‐day alcohol delivery or consumption patterns.

### 1.1 Alcohol Sales Data

Alcohol sales data can be broken into two categories, wholesaler and retailer. Wholesaler sales data are based on sales from an alcohol wholesaler to a retailer, while retail sales are directly to a customer. Wholesaler data can include pricing information, the amount and type of alcohol sold, and the retailer to which it was sold. Retail sales data can include more detailed information about the purchase, including the exact price it was sold to a customer (i.e., accounting for discounts), timing of the transaction, other items purchased in the same transaction, and method of sale (i.e., over the counter, online sale or same‐day delivery). While more limited than retail data, wholesaler sales data have been used to examine impacts of alcohol policies on consumption in Australia [14, 15, 16].

Point‐of‐sale retail sales data obtained through market research companies are available for purchase and use in other international jurisdictions [17], however, these data are not available for direct purchase by public health researchers in Australia. Retail sales data have only been routinely collected by a non‐industry body twice in Australia, both by the NSW Government. The first instance of mandatory sales reporting centred on late night trading venues in the Kings Cross entertainment precinct [18]. More recently though, Liquor and Gaming NSW implemented a framework to mitigate risks associated with same‐day alcohol deliveries to minors and intoxicated individuals on 1 July 2021 [19, 20]. Among the new requirements was the legislated obligation for liquor retailers to report all alcohol delivery sales to Liquor and Gaming NSW [21]. Under this regulation, providers were required to submit six‐monthly sales data within 21 days following 31 December and 30 June each year [19]. While industry supplied retail sales data have been used by the alcohol industry to counter introduction of liquor policies [4, 22], it has not yet been publicly utilised by the government or researchers. This was due to the data being previously inaccessible; it has only recently been made available through a Government Information Public Access request [23]. Establishing the policy health benefit of this data is of high importance, as mandatory retail sales data collection from the Kings Cross precinct was ended because it had not been utilised in research and policy development [18].

### 1.2 The Current Study

While previous research has examined drinking behaviours and motivations associated with same‐day alcohol delivery (e.g., [6]), broader market trends and the geographic distribution of alcohol delivery sales in Australia remain largely unexamined outside of industry reports. To date, no study has utilised retail sales data to monitor same‐day alcohol deliveries. This study aims to quantify the nature of the same‐day delivery market across NSW by geographic distribution, volume of alcohol sold per capita, type of liquor sold, portion of overall alcohol consumption and by number of retailers. Further, comparisons will be made to industry data, aimed at assessing the unique value of the mandatory reporting requirement.

## 2 Methods

Ethics approval for this study has been obtained from Curtin University Human Research Ethics Office (HRE2025‐0114).

### 2.1 Context

New South Wales is Australia's most populous state (8.15 million) and its capital, Sydney, is Australia's most populous city. The NSW government legislated that all same‐day alcohol delivery providers were required to report their delivery data from 1 July 2021 [21]. Australians annually consume approximately 10.46 L of pure alcohol per capita (aged 15 years or older) [24].

### 2.2 Retail Sales Data

An application was made under the *Government Information (Public Access) Act* 2009 to the Department of Creative Industries, Tourism, Hospitality and Sport on 20 September 2024. All data that had been collected as part of the alcohol same‐day delivery sales requirement (s107H; Liquor Regulation 2018) were requested. The request noted that a censored version of the data would be acceptable (e.g., censoring the names of vendors), as commercial and privacy requirements had previously been a concern preventing the publication of the data [25]. The decision was made to release a censored version of this data in November 2024 [23].

Eight variables were obtained, the de‐identified provider (reported as provider 1, provider 2 etc.), the reporting period (6 month blocks, January–June and July–December each year), the postcode alcohol was delivered to, and the combined total volume of litres of liquid supplied (i.e., volume of beverage containers) by the following beverage categories: (i) beer, cider, perry and mead; (ii) wine; (iii) spirits; (iv) ready to drink spirits (pre‐mixed); and (v) total alcohol. All entries were self‐reported, data obtained ranged from July 2021 to June 2024. Any same‐day sale of alcohol is required to be reported and included in this dataset, including those from grocers or restaurants that also contain food items, there is no record of what other items were included in each delivery.

A government review of the mandatory reporting policy outlined, that while Liquor and Gaming NSW were sure that the major delivery providers had submitted the required data, they were unable to confirm whether all providers had complied with the policy [26]. A compliance breach can only be detected if a complaint is made, as Liquor and Gaming NSW do not undertake any compliance operations in relation to reporting requirements for this data, including verification of reported data accuracy [26]. No specific definition of major delivery provider was given, however these likely include large retailers which operate their own delivery services (i.e., Dan Murphy's) and delivery services which service many retailers (i.e., Uber Eats, DoorDash). Deliveries provided by delivery services were grouped, sales from the same retailer in multiple locations were grouped, although it is unclear whether sales from different retailers owned by the same parent company were grouped or reported separately. Further, Liquor and Gaming NSW have confirmed that their data is often sourced from delivery services and that they do not trace these back to the retailer of origin, meaning the data is a mix of delivery service providers and alcohol retailers.

### 2.3 Estimating Litres of Pure Alcohol

Litres of liquid reported did not include alcohol content; alcohol content was estimated using the following methods so that litres of pure alcohol could be calculated to allow comparisons across beverage types:

1. Beer, cider, perry and mead were all treated as beer for the purposes of estimation; this decision was made for simplicity and because beer is the most consumed liquor type in this category by a substantial margin [24]. Beer was divided based on the rounded estimated proportion of the market that consume low strength beer (3%), mid strength beer (25%) and full‐strength beer (72%) [24], these fractions were then multiplied by the average amount of alcohol content in each beer category (2.41%, 3.44% and 4.60% respectively).
2. Wine was divided into three categories based on rounded market share, white (45%), red (39%) and sparkling/other wine (16%) [24]. These fractions were then multiplied by the average amount of alcohol content in each wine category (12.44%, 13.70% and 11.20%); ‘other’ wine was treated as sparkling wine due to substantial market share [27].
3. Spirits were treated as having 40% alcohol content [28].
4. Ready to drink spirits were treated as having 6% alcohol content [28].

### 2.4 Population Data

Postcode level enumerated population data were obtained from the 2021 census; people aged 15 years or older were used in per capita estimates [29]. City and state level residential population estimates were obtained from the Australian Bureau of Statistics [30].

### 2.5 Industry Data

Retail Drinks Australia is the main lobby group for liquor retailers. In 2023 the RDA published a commissioned report examining 2021–22 transaction level retail sales data from retailers accounting for 70% of the market [4]. This data relies on the alcohol industry to have provided accurate data and on their estimate of market share to be accurate. This report included a heat map of transactions across Australian capital cities with postcodes grouped by quintile; data from the Sydney heat map were extracted for comparison to government collected data. Correlation will be used to compare industry data to government collected sales data, by postcode.

### 2.6 National Estimates of Alcohol Consumption

The Australian Institute of Health and Welfare provides national estimates for apparent alcohol consumption each financial year [24]. This dataset is derived from tax revenue and surveys. Beer, spirits and pre‐mixed drinks are taxed based on alcohol content in Australia; as such, estimates in this dataset are derived directly from reporting to the Australian Taxation Office. As wine is taxed on its value rather than alcohol content, the wine consumption estimate is derived through a combination of historic surveys with large wine producers, tax revenue from the sale of wine, and changes in the consumer price index value of wine [24]. Estimates are reliant on tax collection being an accurate reflection of alcohol consumed, noting that wine estimates are likely less accurate as they rely on difficult‐to‐verify assumptions about the wine market. National per capita estimates of pure alcohol were compared to per capita alcohol same‐day delivery sales to establish an estimate for the amount of alcohol consumption same‐day deliveries account for. State and city level estimates are not produced; as such, it was assumed that these would reflect national estimates.

## 3 Results

A total of 49 unique retailers were identified in the dataset, Table 1 shows the number of retailers that reported sales each period, the number of postcodes where sales were reported, and litres of pure alcohol sold. Alcohol was delivered to 513 postcodes across the entire period of 612 possible postcodes. Only eight retailers reported sales in each recording period. Four retailers accounted for 89% of total pure alcohol reported across the period (1,718,637 of 1,956,199 L), each of these four large retailers reported across the entire period. These four retailers had coverage of 319 (213–297) of the 513 postcodes where alcohol was reportedly delivered. There was no clear pattern in sales over time and a large amount of variability by liquor type. Overall reported sales of pure alcohol declined across the period.

**TABLE 1:** Self‐reported alcohol sales, number of retailers reporting and postcodes sold to, by period.

Self‐reports	Reporting period					
	July–December 2021	January–June 2022	July–December 2022	January–June 2023	July–December 2023	January–June 2024
Retailers	23	23	22	16	17	16
Postcodes	388	439	454	452	430	431
Litres of pure alcohol sold as						
Beer	81,318	95,752	75,232	63,786	65,912	63,784
%	20%	31%	25%	25%	17%	22%
Wine	168,079	68,446	118,142	95,792	104,096	116,913
%	40%	22%	40%	37%	26%	41%
Spirits	115,407	124,480	79,181	70,192	196,887	86,326
%	28%	41%	27%	27%	50%	30%
Pre‐mixed	50,859	17,076	25,516	27,026	26,595	19,400
%	12%	6%	9%	11%	7%	7%
**Total**	**415,663**	**305,754**	**298,071**	**256,797**	**393,490**	**286,423**

**FIGURE 1:**
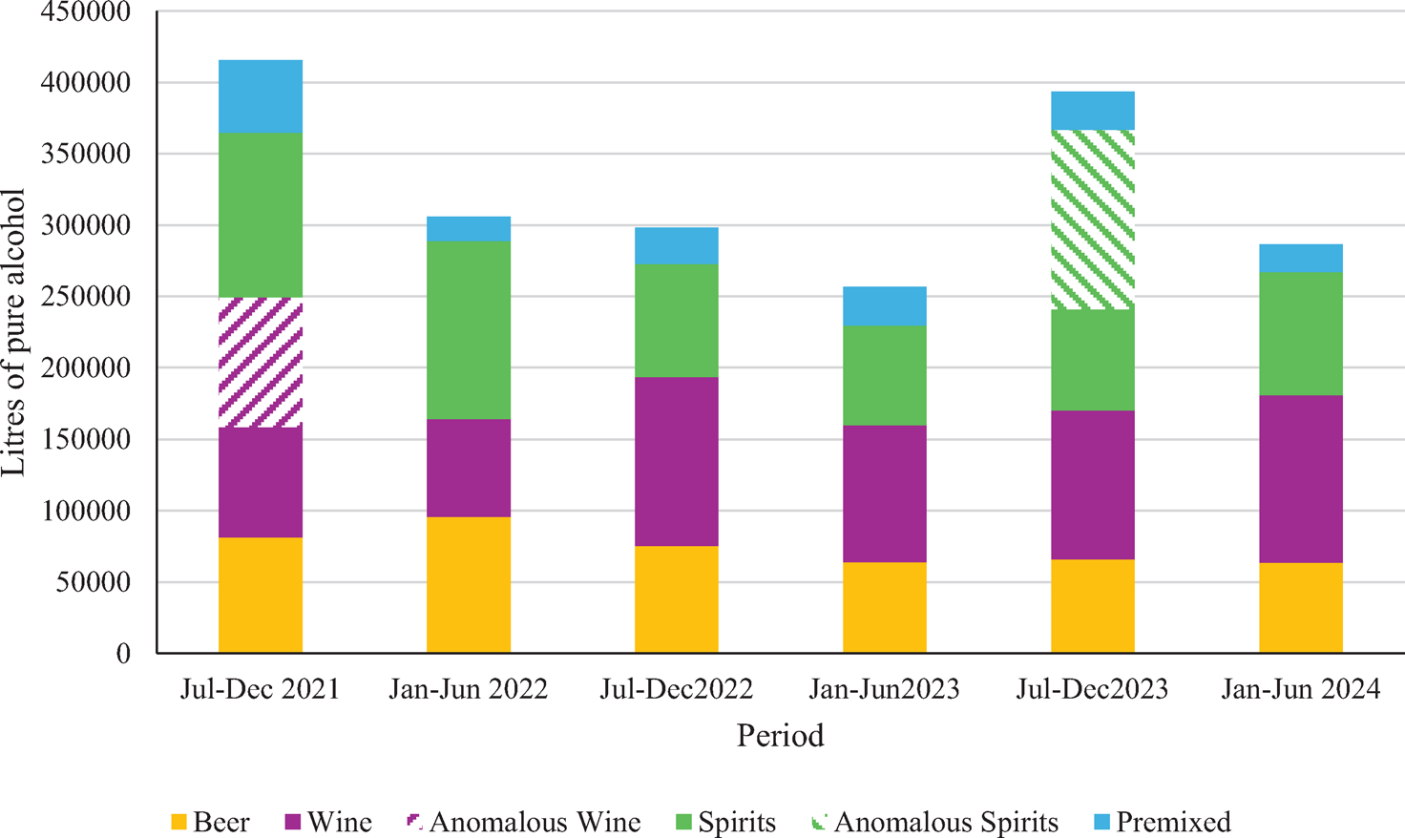
Same‐day delivery sales by liquor category and period.

The substantial increase in spirits sales in July–December 2023 can be attributed to one retailer; this retailer accounts for 9% of all pure alcohol sold across the dataset, reports in each period, but accounted for 46% of pure alcohol sold in July–December 2023. It is unclear whether this is due to an error in reporting as examination of the data shows that the rise in spirits sales occurs across many postcodes, indicating that it is not a single‐entry error. Further, the two highest spirits sales in single postcodes are not attributable to this retailer or period. While less prominent, wine sales in July–December 2021 present a similar anomaly. These can be attributed to high sales reported by one retailer, which accounted for 54% of wine sales in that period but only accounted for 7%–27% in other periods. As with the spirits anomaly, these sales occurred across many postcodes and a separate retailer reported higher sales in a single postcode in the same period. Figure 1 illustrates how pure alcohol sales fluctuated across the period, accounting for the anomalous wine and spirits sales.

**FIGURE 2:**
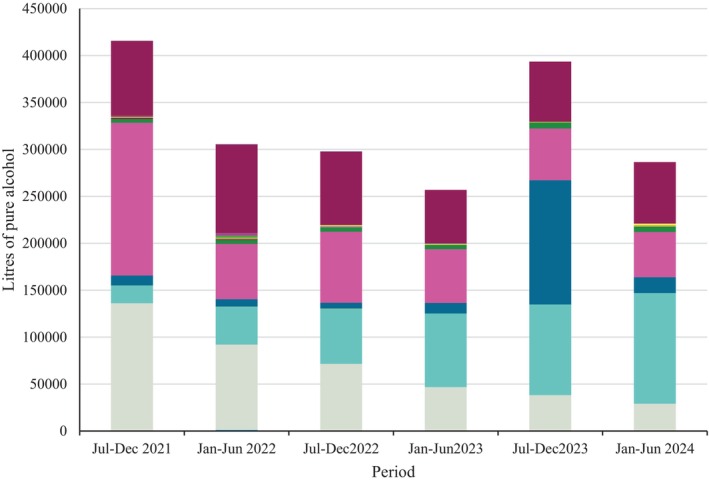
Litres of pure alcohol sold by each retailer and period (*n* = 48). Each colour indicates a different retailer.

Figure 2 shows total pure alcohol sold by each retailer across each period. While the largest retailers are clear throughout the period, there is substantial fluctuation between periods. Figures S1–S4 show this distribution by liquor category.

### 3.1 Geographic Distribution

**FIGURE 3:**
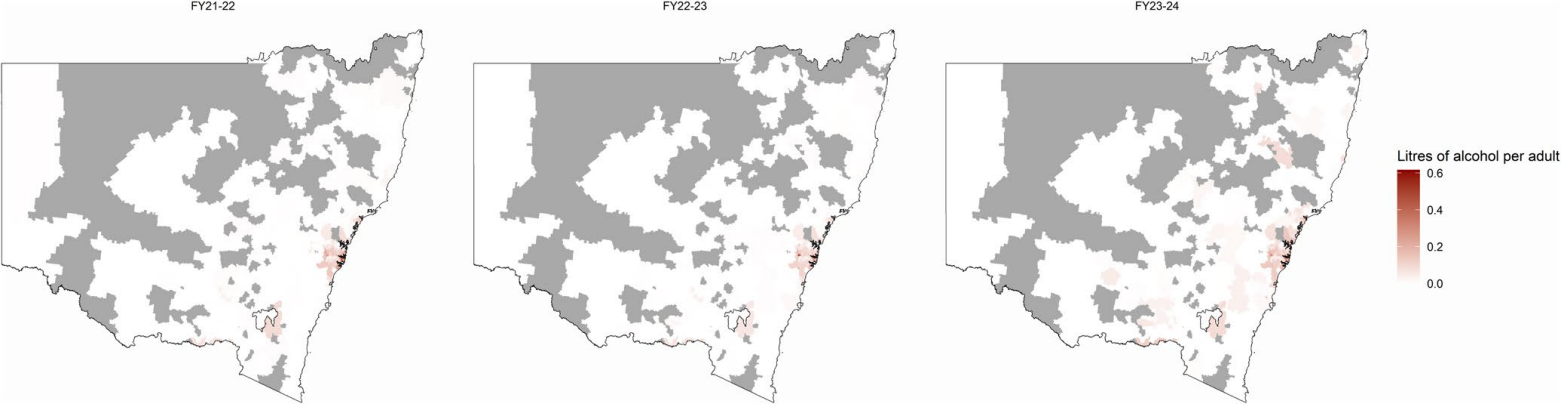
Annual per capita alcohol sales through same‐day delivery retailers across New South Wales, by postcode. Grey areas indicate no data.

Heat maps were utilised to demonstrate the geographic distribution of pure alcohol sales across the state and within the Greater Sydney region (see Appendix A for postcodes). These were condensed to financial years to allow for annual comparisons. The Greater Sydney region accounts for 90%, 89% and 80% of all alcohol sold through same‐day delivery in each financial year. The proportional increase in reporting outside of Sydney can be seen in Figure 3, this resulted in an absolute increase in sales outside of Greater Sydney, from 73,379 L of pure alcohol to 133,749 L. Statewide sales per capita were 0.09 L of pure alcohol in 2021–22, 0.07 in 2022–23, and 0.08 in 2023–24.

**FIGURE 4:**
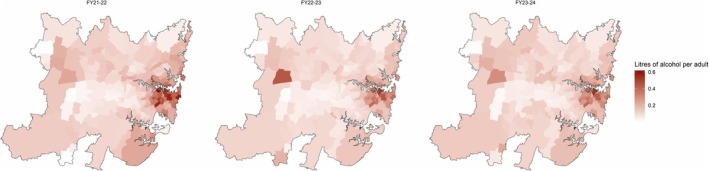
Annual per capita alcohol sales through same‐day delivery retailers across Greater Sydney, by postcode.

Within the Greater Sydney region, alcohol sales through same‐day deliveries were largely concentrated within the city central business district and surrounding areas. As shown in Figure 4, pure alcohol sales per capita saw a slight decline across the whole period, with the most substantial declines in areas with the highest levels of sales. Sales increased in 41 postcodes (18.47%) across the period, 34 of which were in the lower half of pure alcohol sales per capita in the first financial year, all of which had < 0.31 L of alcohol per capita delivered in the final year. City wide per capita consumption was 0.13 L of pure alcohol in 2021–22, 0.10 in 2022–23, and 0.11 in 2023–24.

### 3.2 Comparison to Industry Data

**FIGURE 5:**
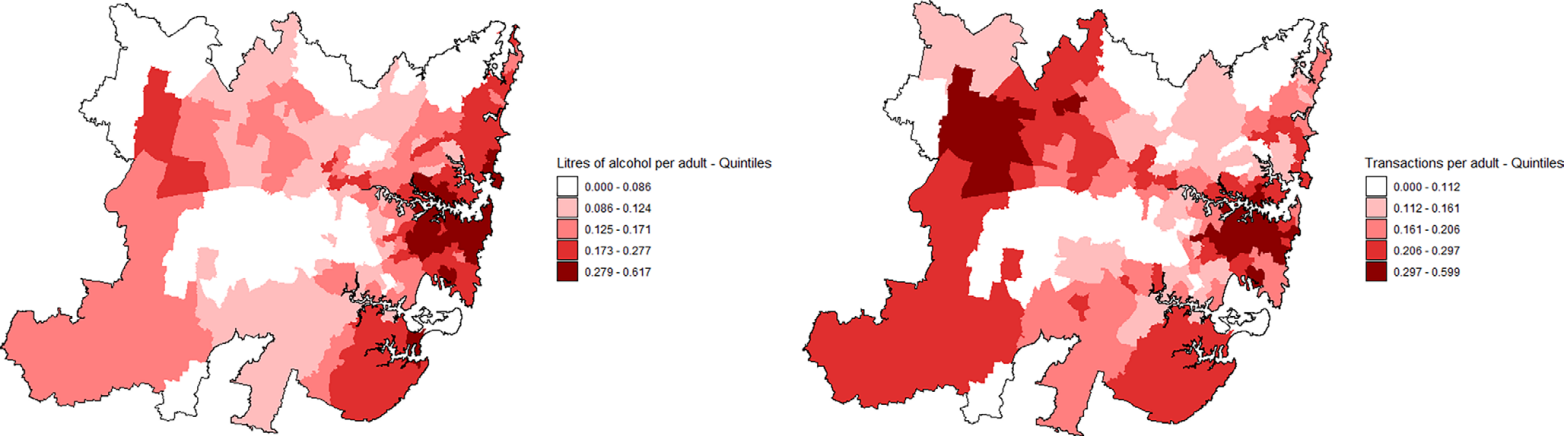
Self‐reported same‐day delivery sales (left) and industry reported transactions (right) in Greater Sydney per capita, by quintile, 2021–2022 financial year.

Pearson correlation was used to examine the association between transactions quintiles extracted from Figure 13 in the alcohol industry report [4] and government collected data. This showed that postcode‐level transactions per capita and per capita consumption quintiles were strongly correlated (*r* = 0.83, *p* < 0.001, 95% confidence intervals 0.78–0.87). This distribution of transactions and per capita consumption is remarkably similar, as shown in Figure 5, suggesting that on average, over a litre of pure alcohol was delivered per transaction.

### 3.3 Comparison to National Estimates

National per capita estimates of total pure alcohol consumption by the AIHW were compared to the same‐day delivery sales data collected by the NSW Government to estimate the portion of total alcohol consumption same‐day delivery sales account for. Table 2 shows that same‐day delivery sales account for less than a percent of alcohol consumption across the entire state, and around 1% of alcohol consumed in Sydney. The portion of spirits consumed through same‐day delivery services was consistently greater than other liquor types but remained below 3% of all spirits consumption.

**TABLE 2:** Comparison of national estimate of litres of pure alcohol consumed per capita to same‐day delivery sales of pure alcohol per capita, by financial year and liquor type.

	Financial year		
	2021–22	2022–23	2023–24
National estimate of all alcohol consumption			
Beer	3.35	3.27	3.12
Wine	4.57	4.42	4.15
Spirits	1.79	1.71	1.51
Pre‐mixed	0.76	0.79	0.76
Total	10.75	10.46	9.76
Same‐day delivery New South Wales			
Beer	0.02	0.02	0.02
%	0.60%	0.61%	0.64%
Wine	0.03	0.03	0.03
%	0.66%	0.68%	0.72%
Spirits	0.03	0.02	0.03
%	1.68%	1.17%	1.99%
Pre‐mixed	0.01	0.01	0.01
%	1.32%	1.27%	1.32%
Total	0.09	0.07	0.08
%	0.84%	0.67%	0.82%
Same‐day delivery Sydney			
Beer	0.03	0.02	0.02
%	0.90%	0.61%	0.64%
Wine	0.04	0.04	0.04
%	0.88%	0.90%	0.96%
Spirits	0.04	0.03	0.04
%	2.23%	1.75%	2.65%
Pre‐mixed	0.01	0.01	0.01
%	1.32%	1.27%	1.32%
Total	0.13	0.10	0.11
%	1.21%	0.96%	1.13%

*Note:* All percentages are portion of national estimate.

## 4 Discussion

This study aimed to examine the nature of the same‐day alcohol delivery market in NSW, utilising retailer self‐reported sales data mandated by NSW liquor regulations. Same‐day delivery service use was largely confined to the Greater Sydney region, accounting for > 80% of all same‐day delivery sales throughout the period. Alcohol sold through same‐day delivery services appears to make up a small fraction of all alcohol consumed in Australia. Our data suggests that the total amount of alcohol reported accounts for < 2% of annual per capita alcohol consumption estimated by the Australian Institute of Health and Welfare [24], same‐day delivery sales likely accounted for < 7% even in postcodes with the highest service utilisation. This finding is around what might be expected from industry reports that same‐day delivery services account for 45% of all online purchases, which make up 13% of all retail alcohol sales (~6% of the market [4]). Alcohol sold from off‐licence retailers makes up roughly 80% of the alcohol consumed in Australia [31], meaning same‐day delivery should make up about ~5% of all alcohol consumption (75% other off‐licence, 20% on‐licence). However, these industry estimates are likely based on the number of sales rather than pure alcohol content. The proportion of overall alcohol consumption that same‐day delivery accounts for should not be used as the sole indicator for the potential harms it presents, how the alcohol is consumed is also an important factor for determining harm [6, 11]. Overall, there appeared to be a decline in the amount of alcohol sold through same‐day delivery services across the period. Although it is difficult to know whether this trend represents a real shift in drinking or issues with data collection. The unique sales data obtained does provide further, limited, insights into the nature of alcohol consumption undertaken through same‐day deliveries.

### 4.1 Overarching Issues

Any interpretation of the data must account for the substantial issues present in the data collection process and that are apparent within the data. Liquor and Gaming NSW are limited in their abilities to ensure all liquor retailers are meeting their reporting obligations as retailers are not differentiated by whether they engage in same‐day delivery or not [26]. Large retailers are clearly captured in the data, and the parallels with data from the industry report are promising [4]. However, there are clear anomalies in the data, substantial fluctuations in liquor sales by category and retailer that are unlikely to be representative of real‐world trends. Without substantial changes to the processes involved in data collection, including error checking and basic data cleaning, these data cannot be considered a valid representation of real‐world trends. Further, more detailed data on compliance would assist in understanding how comprehensively these data represent actual sales.

### 4.2 Liquor Category and Purchase Volume

The distribution of liquor categories observed in same‐day delivery sales data does not match that which would be expected based on national consumption trends [24]. Wine is the most heavily consumed liquor type in Australia, at 4.55 L of pure alcohol per capita, accounting for 43% of all alcohol consumed in Australia. In the same‐day delivery data, wine consumption ranges from 22% to 41% of alcohol sales; beer and cider are likewise underrepresented, at 3.55 L per capita; these beverages account for 34% of alcohol consumed in Australia, but only account for 17%–31% of same‐day delivery sales. Spirits sales only account for 1.71 L per capita and 16% of the alcohol consumed in Australia, while accounting for 28%–50% of same‐day delivery sales. There is no apparent reason to think that these inflated spirit sales are entirely artificial, and examination by retailer demonstrates that this is not simply due to high reporting by one provider every year (see Figure S3). This indicates that spirits are likely genuinely overrepresented through same‐day deliveries.

When compared to the transaction data extracted from the industry report [4], individual same‐day delivery sales appear to contain a considerable amount of alcohol. Comparison between the two datasets suggests that, on average, each transaction contains more than a litre of pure alcohol. This may be attributable to the relatively high volume of spirits sold through same‐day delivery services, as many spirits products can contain over a litre of liquid on their own, 40% of which will be pure alcohol. Large purchases also align with results from surveys, which indicate that heavier alcohol consumers were more likely to use the services and make large purchases [11]. It is also notable that spirits sales in Australia have spiked following the COVID‐19 pandemic and the growth of same‐day delivery services [24].

### 4.3 Strengths and Limitations of the Mandatory Same‐Day Delivery Sales Data Collection

The data collected under the mandatory sales reporting requirement is very limited, especially when compared to data used to inform publicly available industry reports and retail sales data collections undertaken in previous years in Kings Cross, NSW. Categorisation by volume of liquid by liquor type rather than alcohol content is a substantial limitation of this dataset compared to equivalent datasets; Kings Cross data was collected by alcohol content [18] and international examples utilising market research company data often include in depth brand information [32]. Data from the industry report can differentiate between age and gender of each customer, length of the delivery, cost and size of the delivery, and track repeat customers [4]. Speed of delivery is an important issue with clear implications for harm [6, 7, 11], the industry report suggests that rapid deliveries only comprise a small portion of all sales (< 15%; [4]). Although it is important to note that these disproportionately account for sales made within late night hours [4], where alcohol‐related harm is most common [33]. This nuance is lost in the aggregated data collected by the NSW Government; however, it is clear that the same retailers that informed the industry report [4] are represented in the NSW sales data [26]. Changes between retailers in the current dataset may represent changes in service delivery provider rather than changes to specific retailers, due to how the data is collected.

High quality data are already being collected by the industry and used to inform public debate on same‐day delivery legislation [4]. Government needs to enforce the requirement of industry mandatory reporting on home delivery data and make the data available so that these data can be used to inform public health decisions and public policy without first being interpreted by vested interests. Liquor and Gaming NSW do not publicly release or utilise the sales data they currently collect, one reason why their Kings Cross data collection ceased [18]. As an example, wholesaler sales data collected in the Northern Territory, which is publicly released, have been used to monitor alcohol consumption, independently evaluate policy impacts, and support evidence‐informed public discussion that engages with community organisations [14, 34].

Retail sales data collection provides benefits over wholesaler data collection, however the current delivery data in NSW is limited due to insufficient detail and a lack of mechanisms in place to allow for the data to be utilised. Ideally, data collection should expand beyond same‐day delivery services and encompass all alcohol sales within NSW. Other jurisdictions should also consider implementing retail sales data collection either of home delivery or all alcohol sales, because of the clear benefits that well collected data provide in terms of monitoring and evaluation. Of note, industry reports indicate that per capita alcohol consumption through same‐day delivery services in Melbourne is substantially higher than in Sydney [4]. Deaths have been attributed to same‐day delivery use in both cities [8, 35], however, there has been no attempt in Melbourne to quantify and therefore understand this issue. Ideally, data collection would be mandatory with easily enforceable fines for non‐compliance, as compliance has historically waned when fines could not be enforced [18]. Data should be collected directly from individual retailers stores, and verified using data obtained from alcohol wholesalers [14]. The value of point of sales data is in its specificity, such data should include the following by sale: location, liquor category, alcohol content, time of sale, method of sale (i.e., delivery, instore), time from order to delivery where relevant, and size of overall purchase.

## 5 Conclusion

The release of the same‐day delivery sales data gives unprecedented insight into the scope and nature of the same‐day delivery market in NSW, which appears to make up only a small portion of the overall alcohol market. However, this insight is largely undermined by the lack of compliance enforcement or quality controls that would be necessary to ensure the validity of the data. Allowing data collection to be aggregated by delivery service provider, rather than store or chain specific data further undermines the utility of the data; taking the obligation away from retailers to report their own sales only creates further uncertainties for ensuring compliance. Sales data collected by the NSW Government are a step in the right direction, but more needs to be done to ensure data collection is fit for purpose. It is apparent that more detailed data are readily available; policy should be adjusted to make better use of existing industry data in order to better prevent harm. Alcohol consumed through same‐day delivery services makes up a small fraction of the market; substantially improved data collection techniques should be expanded to include all retail sales. Further utilisation in combination with demographic, justice, and health data is essential to better understand the impact same‐day alcohol delivery has on alcohol‐related harms across the population.

## Author Contributions

Each author certifies that their contribution to this work meets the standards of the International Committee of Medical Journal Editors.

## Funding

This work was supported by the Australian Research Council (FT210100656, DE250100685); National Health and Medical Research Council Investigator Fellowship (1174630); and Western Australian Future Health Research and Innovation Fund (WANMAEL2024‐25/9).

## Conflicts of Interest

The authors declare no conflicts of interest.

## Supporting information


**Supplementary Figures S1‐4:** dar70142‐sup‐0001‐Data.docx.

## Data Availability

The data that support the findings of this study are available from the corresponding author upon reasonable request.

## Appendix A Greater Sydney Region Postcodes, Based on Figure 13 of the Industry Report [4]

2000, 2006, 2007, 2008, 2009, 2010, 2011, 2015, 2016, 2017, 2018, 2019, 2020, 2021, 2022, 2023, 2024, 2025, 2026, 2027, 2028, 2029, 2030, 2031, 2032, 2033, 2034, 2035, 2036, 2037, 2038, 2039, 2040, 2041, 2042, 2043, 2044, 2045, 2046, 2047, 2048, 2049, 2050, 2052, 2060, 2061, 2062, 2063, 2064, 2065, 2066, 2067, 2068, 2069, 2070, 2071, 2072, 2073, 2074, 2075, 2076, 2077, 2079, 2080, 2084, 2085, 2086, 2087, 2088, 2089, 2090, 2092, 2093, 2094, 2095, 2096, 2097, 2099, 2100, 2101, 2102, 2103, 2104, 2105, 2106, 2107, 2108, 2109, 2110, 2111, 2112, 2113, 2114, 2115, 2116, 2117, 2118, 2119, 2120, 2121, 2122, 2123, 2125, 2126, 2127, 2128, 2129, 2130, 2131, 2132, 2133, 2134, 2135, 2136, 2137, 2138, 2139, 2140, 2141, 2142, 2143, 2144, 2145, 2146, 2147, 2148, 2150, 2151, 2152, 2153, 2154, 2155, 2156, 2158, 2160, 2161, 2162, 2163, 2164, 2165, 2166, 2167, 2168, 2170, 2171, 2172, 2173, 2174, 2175, 2176, 2177, 2178, 2179, 2190, 2191, 2192, 2193, 2194, 2195, 2196, 2197, 2198, 2199, 2200, 2203, 2204, 2205, 2206, 2207, 2208, 2209, 2210, 2211, 2212, 2213, 2214, 2216, 2217, 2218, 2219, 2220, 2221, 2222, 2223, 2224, 2225, 2226, 2227, 2228, 2229, 2230, 2231, 2232, 2233, 2234, 2555, 2556, 2557, 2558, 2559, 2563, 2564, 2565, 2566, 2567, 2568, 2570, 2745, 2747, 2748, 2749, 2750, 2753, 2765, 2759, 2760, 2761, 2762, 2763, 2765, 2766, 2767, 2768, 2769, 2770, 2773, 2774, 2777.
