# Supplementary material for: Using Mandatory Sales Reports to Monitor Same‐Day Alcohol Delivery Trends in New South Wales
Source: Drug Alcohol Rev. 2026 Mar 18;45(3):e70142. doi: 10.1111/dar.70142 (PMC12998493; doi:10.1111/dar.70142)
Supplement: Supplementary file 1 — Supplementary Figures S1‐4: dar70142‐sup‐0001‐Data.docx. [file DAR-45-0-s001.docx]

Figure S1. Litres of pure alcohol sold as beer by each retailer and period (n=48).

***Note.*** Each colour indicates a different retailer.

Figure S2. Litres of pure alcohol sold as wine by each retailer and period (n=48).

***Note.*** Each colour indicates a different retailer.

Figure S3. Litres of pure alcohol sold as spirits by each retailer and period (n=48).

***Note.*** Each colour indicates a different retailer.

Figure S4. Litres of pure alcohol sold as premixed by each retailer and period (n=48).

***Note.*** Each colour indicates a different retailer.
